# Effects of early mental state changes on physical functions in elderly patients with a history of falls

**DOI:** 10.1186/s12877-023-04274-6

**Published:** 2023-09-15

**Authors:** Yao Cui, Bo Liu, Ming-Zhao Qin, Qian Liu, Hui Ye, Jian Zhou

**Affiliations:** 1 Department of Geriatrics, Beijing Tongren Hospital, Capital Medical University, No.1 of Dong Jiao Min Xiang, Dongcheng District, Beijing, 100730 China; 2 Department of Otolaryngology Head and Neck Surgery, Beijing Tongren Hospital, Dongcheng District, Capital Medical University, Beijing Institute of Otolaryngology, Key Laboratory of Otolaryngology Head and Neck Surgery (Capital Medical University), Ministry of Education, No.1 of Dong Jiao Min Xiang, Beijing, 100730 China

**Keywords:** Elderly, Mental state, SAS/SDS scores, Physical function, Falls

## Abstract

**Background:**

Fear of falling is a potential consequence for older adults who have experienced a fall. Whether such psychological concerns related to falls, in turn, affect physical function? Especially those who have a history of falling but have not been diagnosed with anxiety, depression, or both. This study aimed to clarify the effects of early psychological changes on the physical function of older patients.

**Methods:**

The 111 participants with falling history were divided into the poor physical function (PPF) group with the Short Physical Performance Battery (SPPB) ≤ 9 and the good physical function (GPF) group with SPPB > 9. Their physical function was assessed through 4-m gait speed (4MGS), five times sit-to-stand test (FTSST), grip strength, and Timed Up and Go tests TUGT. Their mental state was assessed by the self-rating anxiety/depression scale (SAS/SDS).

**Results:**

(1) SAS/SDS scores were negatively correlated with the SPPB score, gait speed, and maximum grip strength (males). (2) Multivariate logistic regression analysis showed that the SPPB score was subject to such independent influence factors: cerebrovascular disease (OR = 11.805; *P* = 0.005), normal ratio of grip strength (OR = 0.046; *P* = 0.016), TUGT (OR = 1.717; *P* < 0.001), and SDS score (OR = 1.154; *P* = 0.008). (3) The area under the ROC curve was 0.699 (0.601, 0.797) for SAS score, with a sensitivity of 0.776 and a specificity of 0.547; the AUC was 0.694 (0.596, 0.792) for SDS score, with a sensitivity of 0.586 and a specificity of 0.755.

**Conclusions:**

In older adults with a history of falls without a diagnosis of anxiety or depression, higher SAS/SDS scores were associated with worse fall-related physical function, and there was a statistically significant correlation between the two. This may indicate a risk of falling again in the future.

## Background

As the world's most populous country, China has experienced rapid population aging in recent years. By the end of 2020, the proportion of people aged 60 and above will have increased by 5.44%, and the proportion of people aged 65 and above will have increased by 4.63%, according to the report of China's National Bureau of Statistics [1]. With increasing age and chronic diseases, older adults will have a decline in strength, reaction time, coordination and balance, resulting in impaired physical function. Physical function is defined as an objectively observable systemic mobile function of the body. It includes not only muscle function but also nervous system function, which is a multidimensional concept [2]. Decreased physical function increases the risk of falls in older adults [3]. The annual incidence of falls in the elderly increases with age and has become a global public health problem [4].

Changes in physical function and mental state are both factors that influence falls. More and more studies have found that the mental state of the elderly changes once they experience a fall [5, 6]. This is often followed by a fear of falling, and in some cases, even anxiety and depression [7]. One study found that depression in older people was associated with increased disability and poorer physical function [8]. Similarly, a review concludes that anxiety disorders in older people are associated with poorer physical health outcomes, including an increased risk of falls, reduced activity, and greater functional impairment [9]. However, it is important to consider whether changes in early mental status may also affect physical function in older individuals. This is particularly important for older people who have not been clearly diagnosed with anxiety and depression, as changes in their mental state often occur gradually. This raises the question of whether early changes in psychological state have already had an impact on physical function, enabling the identification of health problems at an early stage and the implementation of appropriate interventions.

According to the recommendations of the Asian Sarcopenia Working Group (AWGS2019) and the Chinese Expert Consensus on the Diagnosis and Treatment of Sarcopenia in the Elderly (2021) [10, 11], physical function was measured using the Short Physical Performance Battery (SPPB), 4-m walk test, five-time sit-to-stand test (FTSST), grip strength test, and timed up and go test (TUGT). Mental status was assessed using the Self-rating Anxiety/Depression Scale (SAS/SDS). This study aims to investigate the relationship between early mental state and physical function in elderly patients with a history of falls by using objective measures of physical function and mental state assessment. The objective was to study the effect of early changes in mental state on physical function in elderly patients with a history of falls.

## Methods

### Participants

This study was a cross-sectional study, which consecutive enrolled 111 elderly patients who experienced at least one fall within 12 months and were admitted to the Department of Geriatrics of Beijing Tongren Hospital for reasons unrelated to falls between January 1, 2020, and December 31, 2021. All subjects provided written informed consent following the Declaration of Helsinki, and the study protocol was approved by the ethical committee of Beijing Tongren Hospital Affiliated to Capital Medical University (approval no. TRECKY2021-042). Falls, defined as “an unexpected event in which the participants come to rest on the ground, floor, or lower level.” [12].

### Inclusion criteria

(1) Age ≥ 60 years old, (2) able to walk for 30 m independently, (3) non acute attack of cardiovascular and cerebrovascular disease, (4) no history of Parkinson's disease, (5) normal cognitive function and comprehension of questionnaire content, (6) no diagnosis of psychological diseases, SAS/SDS scores < 50, and (7) no sedative or sleeping drugs.

### Exclusion criteria

(1) Age < 60 years old, (2) unable to walk independently, (3) unable to understand and accurately cooperate with the examination, (4) abnormal hearing and vision, (5) in the acute attack of heart, brain, lung and kidney diseases, (6) with definite anxiety, depression and other psychological disorders or SAS and/or SDS score ≥ 50.

The SPPB consists of three parts: Standing Balance, Sitting Up Test, and 4-Meter Walking Speed. The standing balance subtest consists of three progressively more challenging parts, which include the feet-together stand, the semi-tandem stand, and the full tandem stand. The sitting-up test requires participants to cross their arms over their chest and perform five chair stands as quickly as possible. Walking speed was assessed by having participants walk 4 m at their usual pace, starting from a moving position. Points are assigned based on the completion status, and the specific scoring criteria are presented in Table 1. According to Chinese and Asian population standards, an SPPB score greater than 9 is considered normal for physical function [10, 11].

**Table 1:** SPPB score

		Time(s)	Point
Standing balance	feet-together stand	≥ 10	1
		< 10	0
	semi-tandem stand	≥ 10	1
		< 10	0
	full-tandem stand	≥ 10	2
		3–9.99	1
		< 3	0
4-m gait speed		< 4.82	4
		4.82 ~ 6.20	3
		6.21 ~ 8.70	2
		≥ 8.71	1
		unavailable	0
FTSST		< 11.19	4
		11.20 ~ 13.69	3
		13.70 ~ 16.69	2
		16.70 ~ 60	1
		> 60 or unavailable	0
SPPB score = balance point + gait speed point + FTSST point			

Standing Balance: ①feet-together stand:1 point for more than or equal to 10 s and 0 point for less than 10 s; ②semi-tandem stand: 1 point for more than or equal to 10 s and 0 point for less than 10 s; ③ full-tandem stand: 2 points for more than or equal to 10 s, 1 point for less than 10 s but more than 3 s, and 0 point for less than 3 s. Total of 4 points

4-m gait speed: 4 point for less than 4.82 s; 3 point for more than 4.82 s and less than 6.20 s; 2 point for more than 6.21 s and less than 8.7 s;1 point for more than 8.71 s; 0 point for unable to complete the test. Total of 4 points

FTSST: 4 point for less than 11.19 s; 3 point for more than 11.20 s and less than 13.69 s; 2 point for more than 13.70 s and less than 16.69 s;1 point for more than 16.70 s less than 60 s; 0 point for more than 60 s or unable to complete the test. Total of 4 points

The total SPPB score is obtained by adding the three parts of the point. Total of 12 points

A total of 111 patients were enrolled according to the inclusion and exclusion criteria. According to the SPPB score, 58 cases were divided into the poor physical function (PPF) group with SPPB ≤ 9, and 53 cases were divided into the good physical function (GPF) group with SPPB > 9.

### Medical history collection and evaluation

1. Clinical data: All patients were tasked to measure/calculate and document their basic clinical data, e.g., age, sex, body height, body weight, body mass index (BMI), underlying condition, and Multiple medication was defined as the use of five or more medications.
2. Laboratory test: Blood tests were run to determine the levels of their hemoglobin (Hgb), albumin (ALB), hemoglobin A1C (HbA1C), serum creatinine (SCr), blood urea nitrogen (BUN), uric acid (UA), total cholesterol (TC), triglyceride (TG), high-density lipoprotein cholesterol (HDL-C), and low-density lipoprotein cholesterol (LDL-C).
3. SAS/SDS: Mental state was evaluated using the self-rating anxiety scale (SAS) and the self-rating depression scale (SDS) [13, 14], respectively. Patients were asked to truthfully complete each of the 20 quantitative items according to their conditions within the previous week on the following 4-point scale: ‘1’means ‘None or a little of the time’, ‘2’means ‘Some of the time’, ‘3’means ‘A good part of the time’, or ‘4’means ‘Most or all the time’. The standardized score was obtained by multiplying the raw score by 1.25 and taking the integer part of the result. A cut-off value of 50 points was applied [15–17]. A score of less than 50 was included in this study.
4. Physical function: The physical function was assessed through Standing balance, 4-m gait speed (4MGS), FTSST, grip strength, and Timed Up and Go tests (TUGT).
  4.1 Standing balance: all patients were asked to complete a feet-together stand, semi-tandem stand, and full-tandem stand, recording the completion time (s) and assigning points based on time results (see Table 1).
  4.2 4MGS: All Participants were required to walk for 6 m, record the time (s) from the second meter to the fifth meter, and calculate the 4-m walking speed (m/s). Simultaneously assign points based on time results (see Table 1).
  4.3 FTSST: Choose a stable chair with a back and a height of 44 cm and use a stopwatch to time it. The subjects were asked to stand with their feet on the ground, with their backs not against the back of the chair, and to cross their hands over their chests. After hearing the order to begin the test, they were asked to complete five standing and sitting movements as quickly as possible. The time for the patient to complete the five standing and sitting movements was recorded (s). Simultaneously assign points based on time results (see Table 1).
  4.4 TUGT: Also, choose a stable chair with a back and a height of 44 cm and use a stopwatch to time it. All patients were recorded standing up from a chair, walking 3 m at their usual pace, turning around, walking back to the chair, and sitting down again with their back against the chair (s).
  4.5 Grip Strength was measured using a grip dynamometer (WCS-100 electronic grip dynamometer, China). Participants stood in a quiet state and held the grip machine at maximum strength for 2 times, recorded the maximum value and calculated the normal ratio of grip strength (male ≥ 26 kg/female ≥ 18 kg was normal) [9].
5. aCCI: All patients were recorded for their diagnosis and comorbidities, and calculated on a comorbidity index according to the age-adjusted Charlson Comorbidity Index (aCCI) (see Table 2).

**Table 2:** The age-adjusted Charlson comorbidity index

Score	Comorbidity condition
1	Myocardial infarction(MI)
1	Congestive heart failure(CHF)
1	Peripheral vascular disease
1	Cerebrovascular disease or transient ischemic attack(TIA)
1	Dementia / Alzheimer's disease
1	Chronic obstructive pulmonary disease / asthma
1	Connective tissue disease
1	Peptic ulcer disease
1	Mild liver disease
1	Diabetes without end-organ damage
2	Hemiplegia
2	Moderate/severe chronic kidney disease
2	Diabetes with end-organ damage
2	Solid tumor without metastasis (exclude if > 5 years from diagnosis)
2	leukemia
2	lymphoma
3	Moderate/severe liver disease
6	Metastatic solid tumor
6	Acquired Immune Deficiency Syndrome(AIDS)
Age adjustment (years) for each decade after 40 years, add 1 point to total score, maximum 4 points	
1	50–59
2	60–69
3	70–79
4	≥ 80

A score of 1 was assigned for congestive heart failure, myocardial infarction, cerebrovascular disease, dementia, peripheral vascular disease, connective tissue disease, chronic obstructive pulmonary disease (COPD), mild liver disease, ulcer disease, or diabetes mellitus without end-organ damage

A score of 2 for moderate-to-severe chronic kidney disease, hemiplegia, solid tumor, diabetes with end-organ damage, lymphoma, or leukemia

A score of 3 for moderate-to-severe liver disease, and a score of 6 for acquired immunodeficiency syndrome and metastatic solid tumors

For patients who over 40 years old, the cumulative score was 1 point for each additional 10 years of age

### Statistical analyses

Statistical analysis of the data was performed using SPSS (version 22.0). The Shapiro–Wilk test was used to test the normality assumption of continuous variables. All continuous variables with normal distribution were presented as mean (standard deviation) (SD), and non-normally distributed continuous variables were summarized as median (25th-75th). Student's t-test was used for normally distributed variables when comparing continuous variables between the two groups, and Mann–Whitney U test was used for non-normally distributed variables. Categorical variables were expressed as percentages and analyzed between groups using the Pearson chi-squared test (χ 2). Spearman correlation was used to understand the relationship between physical function and mental status. Binary logistic regression was used for multivariate analysis with the influencing factors of physical function, presented as odds ratios (ORs) with 95% confidence intervals (CI), with physical function as a binary variable. To analyze the predictive power of selected predictors, receiver operating characteristic (ROC) curves were calculated and the area under the curve (AUC) was determined. A *P* < 0.05 was considered statistically significant. All statistical analyses were two-tailed.

## Results

1. Two groups of basic information and general conditions (Table 3)

**Table 3:** Two groups of basic information and laboratory examination

	PPF group (*n* = 58)	GPF group(*n* = 53)	*p*
Basic information			
Age (years)	85.00(81.50, 89.00)	76.00 (70, 83.50)	< 0.001
Male [n (%)]	42 (72.414)	39 (73.58)	0.890
BMI (kg/m2)	24.71 (22.78, 26.45)	23.14 (21.29, 25.64)	0.142
aCCI (score)	7.0 (6.0, 8.0)	5.0 (4.0, 7.0)	< 0.001
Hypertension [n (%)]	46 (79.31)	37 (69.81)	0.280
Coronary heart disease [n (%)]	27 (46.55)	22 (41.51)	0.702
Diabetes [n (%)]	29 (50.00)	28 (52.83)	0.850
Solid tumor [n (%)]	13 (22.41)	5 (9.43)	0.075
Chronic kidney disease [n (%)]	16 (27.59)	13 (24.53)	0.829
Cerebrovascular disease [n (%)]	26 (44.83)	6 (11.32)	< 0.001
Chronic obstructive pulmonary disease [n (%)]	17 (29.31)	7 (13.21)	0.063
Multiple medication history [n (%)]	33 (56.90)	31 (58.49)	0.425
laboratory examination			
Hgb (g/L)	126.00(112.25, 138.00)	129.00 (118.00, 137.50)	0.360
BUN (mmol/L)	6.05 (4.88, 8.15)	5.40 (4.75, 7.95)	0.143
SCr (µmol/L)	77.65 (66.40, 101.98)	77.2 (67.65, 88.18)	0.798
UA (µmol/L)	386.00 (293.70, 438.00)	341.45 (281.03, 386.60)	0.026
ALB (g/L)	36.40 (33.40, 38.70)	37.50 (35.93, 40.53)	0.011
HbA1C (%)	6.25 (5.80, 7.60)	6.15 (5.68, 7.43)	0.315
TG (mmol/L)	1.16 (0.76, 1.49)	1.25 (0.73, 1.96)	0.310
TC (mmol/L)	4.06 (3.51, 4.69)	3.72 (3.03, 5.05)	0.312
HDL-C (mmol/L)]	1.17 (0.97, 1.45)	1.18 (0.98, 1.35)	0.232
LDL-C (mmol/L)	2.21 (1.88, 2.73)	2.07 (1.42, 3.14)	0.675

*BMI* Body mass index, *aCCI* The age-adjusted Charlson Comorbidity Index, *Hgb* Hemoglobin, *BUN* Blood urea nitrogen, *SCr* Serum creatinine, *UA* Uric acid, *ALB* Albumin, *HbA1C* Hemoglobin A1C, *TG* triglyceride, *TC* Total cholesterol, *HDL-C* High-density lipoprotein cholesterol, *LDL-C* Low-density lipoprotein cholesterol

The values are presented as median (interquartile range) or number (%). A *p*-value < 0.05 was considered statistically significant

A total of 111 patients were divided into the PPF group (*n* = 58) and the GPF group (*n* = 53), with a mean age of 80.45 years (ranging from 60 to 99). The study included 81 males and 30 females. There were significant differences in age, aCCI, the cerebrovascular disease and UA in the PPF group were higher than those in the GPF group, whereas ALB in the PPF group was lower than those in the GPF group (P < 0.05). There was no statistical difference in the remaining basic information, medical history, and laboratory parameters (*P* > 0.05).

## 2. Differences in physical function and mental state between the two groups (Table 4 & Fig. 1)

**Table 4:** Differences of physical function and mental state between the two groups

	PPF group(*n* = 58)	GPF group(*n* = 53)	*p*
Gait speed (m/s)	0.68 (0.56, 0.80)	0.97 (0.89, 1.12)	< 0.001
FTSST (s)	14.75 (12.09, 20.18)	11.31 (9.62, 13.33)	< 0.001
TUGT (s)	16.60 (12.64, 21.06)	9.81 (8.44, 11.11)	< 0.001
SPPB (score)	7 (6, 8)	11 (10, 12)	< 0.001
normal ratio of grip strength [n (%)]	55.769%	92.0%	< 0.001
Men grip strength (kg)	23.95 (19.63, 29.40)	31.75 (23.23, 38.08)	< 0.001
Women grip strength (kg)	18.00 (12.40, 21.20)	20.70 (18.38, 23.08)	0.049
SAS (score)	35.00 (31.25, 40.00)	30.00 (27.00, 35.00)	< 0.001
SDS (score)	38.00 (31.25, 42.50)	31.00 (26.25, 35.00)	< 0.001

*FTSST* Five Times Sit- To-Stand Test, *TUGT* Timed Up to Go Test, *SPPB* Short Physical Performance Battery, *SAS* the Self-Rating Anxiety Scale, *SDS* the Self-Rating Depression Scale. The values are presented as median (interquartile range) or number (%). A *p*-value < 0.05 was considered statistically significant

**Fig. 1:**
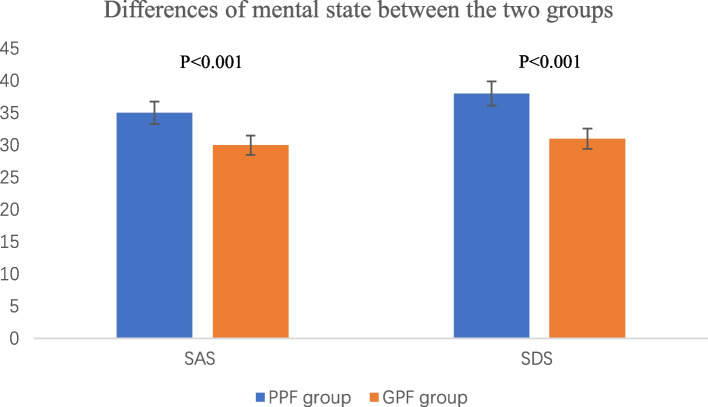
Differences of mental state between the two groups. SAS: the Self-Rating Anxiety Scale; SDS: the Self-Rating Depression Scale. PPF: the poor physical function group; GPF: the good physical function group. The SAS/SDS scores in the PPF group were higher than those in the GPF group with statistically significant differences (*P* < 0.05)

The gait speed, normal ratio of grip strength, and maximum grip strength decreased significantly in the PPF group, compared with the GPF group (P < 0.05). While FTSST, TUGT prolonged significantly in the PPF group, compared with the GPF group (P < 0.05). The SAS/SDS scores in the PPF group were higher than those in the GPF group with statistically significant differences (P < 0.05).

## 3. The correlation between Mental Status and physical function

3.1 Spearman correlation showed the SAS and SDS scores were weak negatively correlated with the SPPB score (*r* = -0.298 *p* = 0.001; *r* = -0.349 *p* = 0.001, respectively) gait speed (*r* = -0.272; *p* < 0.001; *r* = -0.361; *p* < 0.001, respectively), maximum grip strength of male (r = -0.367; *p* < 0.001; *r* = -0.219; *p* = 0.027, respectively). The SAS and SDS scores were not correlated with the FTSST, TUGT, and maximum grip strength of female (*P* > 0.05). (Table 5).

**Table 5:** The correlation between Mental State and physical function

	SAS		SDS	
	r	*p*	r	p
SPPB	-0.298	0.001	-0.349	0.001
Gait speed	-0.272	< 0.001	-0.361	< 0.001
Men grip strength	-0.367	< 0.001	-0.219	0.027
Women grip strength	-0.378	0.043	-0.111	0.565
FTSST	0.032	0.749	0.102	0.231
TUGT	0.133	0.191	0.216	0.066

*SPPB* Short Physical Performance Battery, *FTSST* Five Times Sit- To-Stand Test, *TUGT* Timed Up to Go Test; A *p*-value < 0.05 was considered statistically significant

3.2 Binary logistic regression analysis was conducted with SPPB > 9 as the binary variable. The results showed that the SPPB of the fall-affected elderly were subject to such independent influence factors: the history of cerebrovascular disease (OR = 11.805; *P* = 0.005), normal range of grip strength (OR = 0.046; *P* = 0.016), TUGT score (OR = 1.717; *P* < 0.001), and SDS score (OR = 1.154; *P* = 0.008), after adjusting for age, gender, and BMI (Table 6).

**Table 6:** Binary logistic regression analysis of SPPB in elderly falls

variates	OR	95% CI	*p*
Cerebrovascular disease	11.805	2.119, 65.772	0.005
normal rate of grip strength	0.046	0.005, 0.441	0.016
TUGT	1.717	1.296, 2.273	< 0.001
SDS score	1.154	1.039, 1.282	0.008
Constant	0.000		0.003

*TUGT* Timed Up to Go Test, *SDS* the Self-Rating Depression Scale

A *p*-value < 0.05 was considered statistically significant

**Fig. 2:**
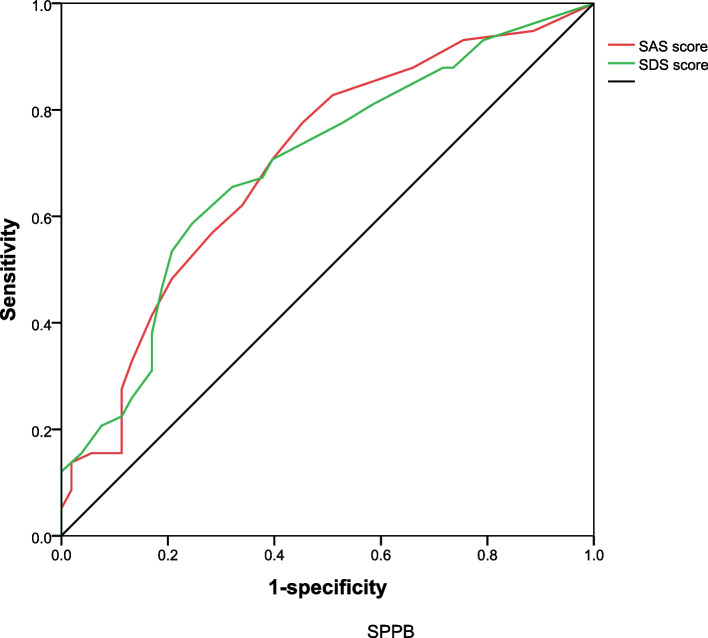
Correlation between mental state and physical function. SPPB: Short Physical Performance Battery; SAS: the Self-Rating Anxiety Scale; SDS: the Self-Rating Depression Scale. The AUC was 0.699 (0.601, 0.797) for SAS score, with a sensitivity of 0.776 and a specificity of 0.547; the AUC was 0.694 (0.596, 0.792) for SDS score, with a sensitivity of 0.586 and a specificity of 0.755

3.3 A receiver operating characteristic (ROC) curve was drawn with SPPB ≤ 9 as a positive rate, the AUC was 0.699 (0.601, 0.797) for SAS score, with a sensitivity of 0.776 and a specificity of 0.547; the AUC was 0.694 (0.596, 0.792) for SDS score, with a sensitivity of 0.586 and a specificity of 0.755 (Fig. 2).

## Discussion

With age, the annual incidence of falls in the older adults also increases. About one-third of older individuals over the age of 65 experience multiple falls in a year, with the annual occurrence of falls in those over 80 years old reaching as high as 50% [18]. Falling can trigger a fear of falling, as well as anxiety and depression [19]. It is also highly significant to analyze the impact of early changes in their psychological state on physical function for fall prevention, particularly among older individuals who have previously experienced falls. In this study, all participants were divided into the PPF (poor physical function) and GPF (good physical function) groups based on their SPPB (Short Physical Performance Battery) score. The age, aCCI, and the incidence of cerebrovascular disease in the PPF group were higher than those in the GPF group. The serum albumin level of the PPF group was lower than that of the GPF group. And the gait speed, normal grip strength rate, and maximum grip strength in the PPF group were all lower than those in the GPF group. The time for FTSST and TUGT in the PPF group was longer than that in the GPF group. It has been suggested that factors such as advancing age, a history of cerebrovascular disease, multiple comorbidities, and deteriorating nutritional status may contribute to a decline in physical function. This decline is primarily characterized by a decrease in walking speed, reduced grip strength, and an increase in the time it takes to complete the FTSST and TUGT tasks.

None of the enrolled patients had a definitive diagnosis of anxiety or depression. The SAS and SDS self-assessment scales were used to evaluate their mental state. The SAS and SDS scores in both groups did not exceed 50 points, indicating that their psychological status showed no significant abnormalities. However, there was a statistically significant difference in SAS and SDS scores between the two groups (*P* < 0.001) (Fig. 1). Additionally, both scores in the PPF group were higher than those in the GPF group, indicating that early changes in psychological state and a tendency towards anxiety or depression occurred with the decline of physical function. An in-depth interview study of older adults with a fear of falling in the United Kingdom also found that older individuals who perceive themselves as being at risk of falling consciously modify their behavior in potentially hazardous activities. The study suggested that participants recognized their vulnerability to falls, which caused them to worry about losing their balance in situations that posed a risk. When the individual perceived that they had control over the subject of their worries, it led to protective adaptations in their behavior. In contrast, when the subject of their worries was perceived to be outside of their control, worries triggered feelings of anxiety, leading to unhelpful changes in behavior [20], including physical dysfunction.

When the activity of the elderly decreases, muscle atrophy is very likely to occur, resulting in a reduction of muscle strength. The grip strength test is a commonly used method for measuring muscle strength in the upper limbs. Additionally, gait speed can indirectly reflect the muscle function in the lower limbs. Therefore, gait speed and grip strength can reflect overall muscle function to some extent, and muscle function is also an important indicator of physical function. In this study, the SAS/SDS scores were found to have a negative correlation with SPPB, gait speed, and maximum grip strength. This indicates that higher SAS and SDS scores were associated with longer time taken to walk 4 m and a greater decline in grip strength. These findings suggest that early changes in mental state may affect muscle function. In recent years, researchers have also discovered a correlation between anxiety and depression, and walking speed and grip strength. Additionally, early intervention for abnormal psychological states can help mitigate the decline of physical function. A cross-sectional study of older hospitalized patients in China found that older patients with low grip strength had an increased risk of depression [21]. According to an analysis of data from the Amsterdam Longitudinal Aging Study, a slow pace was found to be an important factor for depression in older adults [22]. A four-year longitudinal study on Aging in England revealed that neither the presence nor the improvement of depression can delay the decline of grip strength. Therefore, it is crucial to effectively manage depressive symptoms in order to prevent poor physical function [23]. Therefore, early detection and control of the progression of anxiety and depression are of great significance in maintaining physical well-being.

Logistic regression analysis showed that the SAS score was a risk factor for SPPB in elderly falls, but it was not a significant independent risk factor. This may be because anxiety is also influenced by gender, comorbidity, cerebrovascular disease, and other related factors. Older adults may experience anxiety due to previous falls. Consequently, when evaluating physical function, they may unconsciously decrease their pace and limit their range of motion. In daily life, this anxiety may lead to muscle atrophy due to reduced activity, which further reduces physical function and increases the risk of falling. In this study, multivariate logistic regression showed that the SDS score was an independent influencing factor of SPPB. A cross-sectional study of middle-aged women in Singapore found a clear link between physical function and depression. Weak upper and lower body physical performances were associated with depressive and anxiety symptoms [24]. The core symptoms of depression include low mood, loss of interest, and decreased activity [25]. Moreover, older people with early depression may also experience somatization disorders such as shortness of breath, pain, nausea, indigestion, constipation, dysuria, and sleep disturbance. All of these symptoms may decrease physical activity to some extent and result in a decline in physical function. In this study, the positive rate of SPPB ≤ 9 was used to draw the ROC curve (Fig. 2). The area under the curve (AUC) was 0.699 for SAS score (with a sensitivity of 0.776 and specificity of 0.547) and 0.694 for SDS score (with a sensitivity of 0.586 and specificity of 0.755). This indicates that as SAS/SDS scores increase, physical function tends to decrease. Therefore, the SAS/SDS scores have predictive value for assessing the physical function of older individuals with a history of falls.

## Conclusion

The incidence of falls in older adults is higher, and both physical function and psychological state are influential factors in falls. Early anxiety and depression in elderly individuals with a history of falls can further contribute to the decline in physical function. The SAS/SDS scores were negatively correlated with SPPB, gait speed, and grip strength. The higher the scores of SAS and SDS, the worse the physical function. The scores of SAS and SDS had predictive value for the physical function of older individuals with a history of falls. Therefore, early detection and control of the progression of anxiety and depression are of great significance in maintaining overall physical well-being.

## Data Availability

The datasets used in the current study are not publicly available due to them containing information that could compromise research participant privacy but are available from the corresponding author on reasonable request.

## Abbreviations

SPPB: Short Physical Performance Battery
FTSST: Five Times Sit- To-Stand Test
TUGT: Timed Up to Go Test
SAS: Self-Rating Anxiety Scale
SDS: Self-Rating Depression Scale
